# Acute Exercise Improves Insulin Clearance and Increases the Expression of Insulin-Degrading Enzyme in the Liver and Skeletal Muscle of Swiss Mice

**DOI:** 10.1371/journal.pone.0160239

**Published:** 2016-07-28

**Authors:** Mirian A. Kurauti, Ricardo Freitas-Dias, Sandra M. Ferreira, Jean F. Vettorazzi, Tarlliza R. Nardelli, Hygor N. Araujo, Gustavo J. Santos, Everardo M. Carneiro, Antonio C. Boschero, Luiz F. Rezende, José M. Costa-Júnior

**Affiliations:** 1 Department of Structural and Functional Biology, Institute of Biology, State University of Campinas (UNICAMP), Campinas, SP, Brazil; 2 Laboratory of Exercise Physiology, Department of Physical Therapy, University of Pernambuco (UPE), Pernambuco, PE, Brazil; 3 Department of Physiological Sciences, Center of Biological Sciences, Federal University of Santa Catarina (UFSC), Florianopolis, SC, Brazil; 4 Laboratory of Health Sciences, Department of Physiopathology, State University of Montes Claros (UNIMONTES), Montes Claros, MG, Brazil; Universidad Miguel Hernández de Elche, SPAIN

## Abstract

The effects of exercise on insulin clearance and IDE expression are not yet fully elucidated. Here, we have explored the effect of acute exercise on insulin clearance and IDE expression in lean mice. Male Swiss mice were subjected to a single bout of exercise on a speed/angle controlled treadmill for 3-h at approximately 60–70% of maximum oxygen consumption. As expected, acute exercise reduced glycemia and insulinemia, and increased insulin tolerance. The activity of AMPK-ACC, but not of IR-Akt, pathway was increased in the liver and skeletal muscle of trained mice. In an apparent contrast to the reduced insulinemia, glucose-stimulated insulin secretion was increased in isolated islets of these mice. However, insulin clearance was increased after acute exercise and was accompanied by increased expression of the insulin-degrading enzyme (IDE), in the liver and skeletal muscle. Finally, C2C12, but not HEPG2 cells, incubated at different concentrations of 5-aminoimidazole-4-carboxamide-1-β-d-ribofuranoside (AICAR) for 3-h, showed increased expression of IDE. In conclusion, acute exercise increases insulin clearance, probably due to an augmentation of IDE expression in the liver and skeletal muscle. The elevated IDE expression, in the skeletal muscle, seems to be mediated by activation of AMPK-ACC pathway, in response to exercise. We believe that the increase in the IDE expression, comprise a safety measure to maintain glycemia at or close to physiological levels, turning physical exercise more effective and safe.

## Introduction

Insulin action depends on three major physiological processes: insulin sensitivity [1], insulin secretion [2], and insulin clearance [3], and each one of these processes may be influenced by several pathophysiological conditions, such as obesity and diabetes.

Alterations in insulin sensitivity [4–7] and secretion [8–11] have been extensively studied during the last decades; however, less attention has been paid to the study of insulin clearance.

Insulin clearance occurs mainly in the liver due to insulin degradation mediated, primarily, by insulin-degrading enzyme (IDE) [12]. In humans [13–15] and animal models [16–18], obesity reduces insulin clearance, probably due to lower IDE expression and activity in the liver [17, 18]. However, some controversies still remain because higher IDE expression and activity were reported in the liver of obese mice [19, 20]. Despite these discrepancies, several studies have demonstrated that impairment on IDE expression and/or activity is closely related to the onset and development of type 2 diabetes [21–26].

Physical exercise, recommended to obese and diabetic patients, has been show to increase insulin clearance [27, 28] and IDE expression [18, 29] in these patients. These effects contribute to reduce the hyperinsulinemia, often associated with obesity, insulin resistance, and diabetes [15, 30]. Therefore, increased insulin clearance and IDE expression could be another beneficial effect of exercise on the treatment and/or prevention of diseases related to insulin resistance.

In normoinsulinemic lean humans [27, 31, 32] and rats [33], physical exercise also increases insulin clearance, but none of these studies have explored the IDE expression. Here, we found that acute exercise increase insulin clearance probably due to an augmented IDE expression in the liver and skeletal muscle. We also demonstrated that activation of AMP-activated protein kinase (AMPK) might be the mechanism whereby exercise increases IDE expression, in the skeletal muscle, but not in the liver. We hypothesize that the increase of the IDE expression and insulin clearance could be a safety measure to maintain glycemia at or close to physiological levels, turning physical exercise more effective and safe.

## Materials and Methods

### Animals

The 4-week-old male Swiss mice, acquired from the State University of Campinas Facilities, were maintained on a 12 h light-dark cycle at 20–21°C with controlled humidity during the entire experiment. The mice were allowed to feed and drink tap water *ad libitum*, for 8 weeks. All of the experiments were approved by the State University of Campinas Ethics Committee (approval number 1984–1), prior to starting.

### Oxygen consumption (VO_2_) and exercise protocols

**Fig 1:**
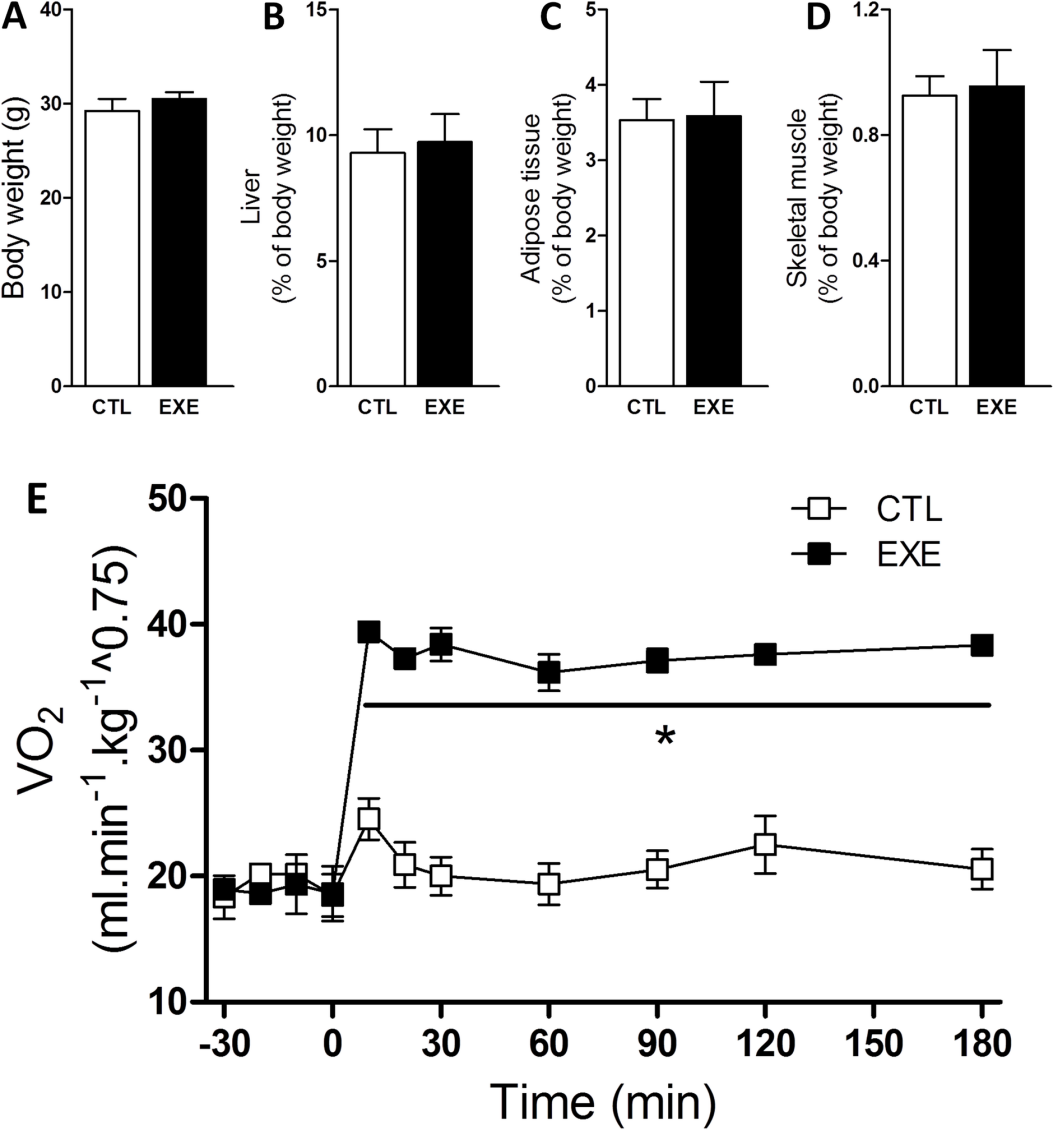
Acute exercise increased VO_2_ without affecting body composition. Total body weight (A), relative liver weight (B), relative perigonadal fat weight (C) and relative gastrocnemius muscle weight (D) were unaltered in mice after a single bout of 3-h exercise on the treadmill under an increased VO_2_ (E). The data are presented as the mean ± S.E.M., n = 3–5. *p≤0.05 *vs* control.

Prior to the acute exercise session, we measured maximum oxygen consumption (VO_2_ max) of all mice (S1 Fig and S1 Table). For the VO_2_ max test, the mice were placed on individual sealed treadmills, with a 25° incline, and attached to a gas analyzer (Oxylet system, Pan Lab/Harvard Appratus, Spain). The treadmill exercise for this test included a warm-up period of 5 min at 10 cm sec^-1^. Subsequently, the treadmill speed was increased by 5 cm sec^-1^ each minute and the oxygen data continuously recorded, at 1 sec intervals, until mice exhaustion, using the METABOLISM Software (Pan Lab/Harvard Instruments, Spain). We defined exhaustion as the point when the mice were unable to keep pace with the set treadmill speed [34]. After 10 days, the mice were randomly assigned to a sedentary control group (CTL), which was limited to typical movement inside the cages, and the exercised group (EXE), which was submitted to an acute exercise on a treadmill inclined at 25° for 3-h at 60–70% of VO_2_ max. During the 3-h of exercise (EXE) or non-exercise (CTL), we measured the VO_2_ of both groups (Fig 1E). All of the experiments described below were performed immediately after the 3-h exercise.

### Intraperitoneal insulin tolerance test (ipITT)

Non-fasted mice received an intraperitoneal bolus of insulin (1 U kg^-1^). The blood glucose was measured using test strips (Accu-Chek Performa II) at baseline (0 min, before receiving insulin) and at 5, 10, 15, 20, 25 and 30 min after the administration of the insulin bolus. Glucose values were converted to natural logarithmic (Ln). The slope was calculated using linear regression (time × Ln [glucose]) and multiplied by 100 to obtain the glucose decay rate constant during the insulin tolerance test (*k*_ITT_, % min^-1^).

### Insulin clearance

The plasma insulin concentrations were also evaluated during the insulin tolerance test, and the insulin clearance was calculated as previously described [35]. The rate constant for insulin loss (insulin decay) was assessed by converting the insulin values to natural logarithmic (Ln); the slope was calculated using linear regression (time × Ln [insulin]); and the results were multiplied by 100 to obtain the insulin decay rate constant (% min^-1^).

### Tissue samples

The mice were killed in a CO_2_-saturated atmosphere, immediately followed by decapitation. We extracted liver and muscle samples from the mice 5 min after an intraperitoneal bolus of 1 U kg^-1^ insulin (Humulin®R, Eli Lilly, São Paulo, Brazil) or saline solution (0.9% NaCl wt/vol). The liver and muscle samples were snap frozen in liquid nitrogen and stored for subsequent protein. After, these tissues were homogenized using a lysate buffer (10 mmol L^-1^ EDTA, 100 mmol L^-1^ Tris base, 100 mmol L^-1^ sodium pyrophosphate, 100 mmol L^-1^ sodium fluoride, 10 mmol L^-1^ sodium orthovanadate, 2 mmol L^-1^ PMSF, 1% Triton X-100 and 1 μg mL^-1^ aprotinin). Pancreatic islets were isolated from the mice immediately after the exercise, as previously described [36].

### Glucose-stimulated insulin secretion (GSIS)

Batches of 10 islets were pre-incubated for 1 h in *Krebs*-Henseleit buffer solution (KHBS) containing 0.5 g l^-1^ of BSA and 5.6 mmol l^-1^ glucose and equilibrated at 95% O_2_ and 5% CO_2_ at 37°C. The medium was discarded, and the islets were incubated for an additional hour in 1 ml of KHBS containing 2.8 mmol l^-1^ or 16.7 mmol l^-1^ of glucose. Subsequently, the supernatant fraction was collected to evaluate insulin secretion, and the remaining islets were homogenized in an alcohol and acid solution to measure the total insulin content by radioimmunoassay [37].

### HEPG2 and C2C12 cells culture

HEPG2 cells (a human liver carcinoma cell line) were culture for 3 days in Dulbecco's Modified Eagle Medium, DMEM (Vitrocell, Campinas, SP, Brazil), enriched with 10% vol./vol. fetal bovine serum (FBS), under a humidified condition with 5% CO_2_ at 37°C. C2C12 cells (a mouse myoblast cell line) were culture in DMEM high glucose (Sigma Aldrich, St. Louis, MO, USA), supplemented with 10% vol./vol. FBS and 1% vol./vol. penicillin-streptomycin, under a humidified condition with 5% CO_2_ at 37°C. After obtain total confluence, the cells were differentiated using DMEM high glucose containing 2% vol./vol. horse serum for 5 days. After that, HEPG2 and differentiated C2C12 cells were incubate at 250, 500 and 750 μmol l^-1^ of 5-aminoimidazole-4-carboxamide-1-β-d-ribofuranoside (AICAR) (TOCRIS Bioscience, Bristol, England, UK), for 3-h. After, the cells were collected in trypsin/EDTA, washed with phosphate-buffered saline (PBS), and homogenized in urea anti-protease/anti-phosphatase buffer for subsequent western blot analysis.

### Western blotting

Western blotting for protein expression and phosphorylation were performed as previously described [38]. The primary antibodies used for Western blotting were as follows: anti-phospho-AMPK and anti-phospho-ACC (Cell Signaling Technology, Boston, MA, USA); anti-IDE, anti-GAPDH, anti-phospho-IR and anti-phospho-Akt (Santa Cruz Biotechnology, Dallas, TX, EUA).

### Statistical analyses

Point-to-point and groups of mice were compared by Student’s *t*-test. Groups from the *in vitro* experiments (HEPG2 and C2C12 cell culture) were compared by one-way ANOVA using the unpaired Tukey’s *post hoc* test (GraphPad Prism 5, La Jolla, CA, USA). Data are presented as the mean ± standard error means (SEM). A value of *p*<0.05 was considered to be statistically significant.

## Results

### Exercise altered metabolic parameters

There was no change in body weight (Fig 1A), liver (Fig 1B), perigonadal fat (Fig 1C), or gastrocnemius weight (Fig 1D) after a single bout of exercise; however, the VO_2_ increased during the experiment (Fig 1E).

### Exercise altered glycemia and insulinemia

**Fig 2:**
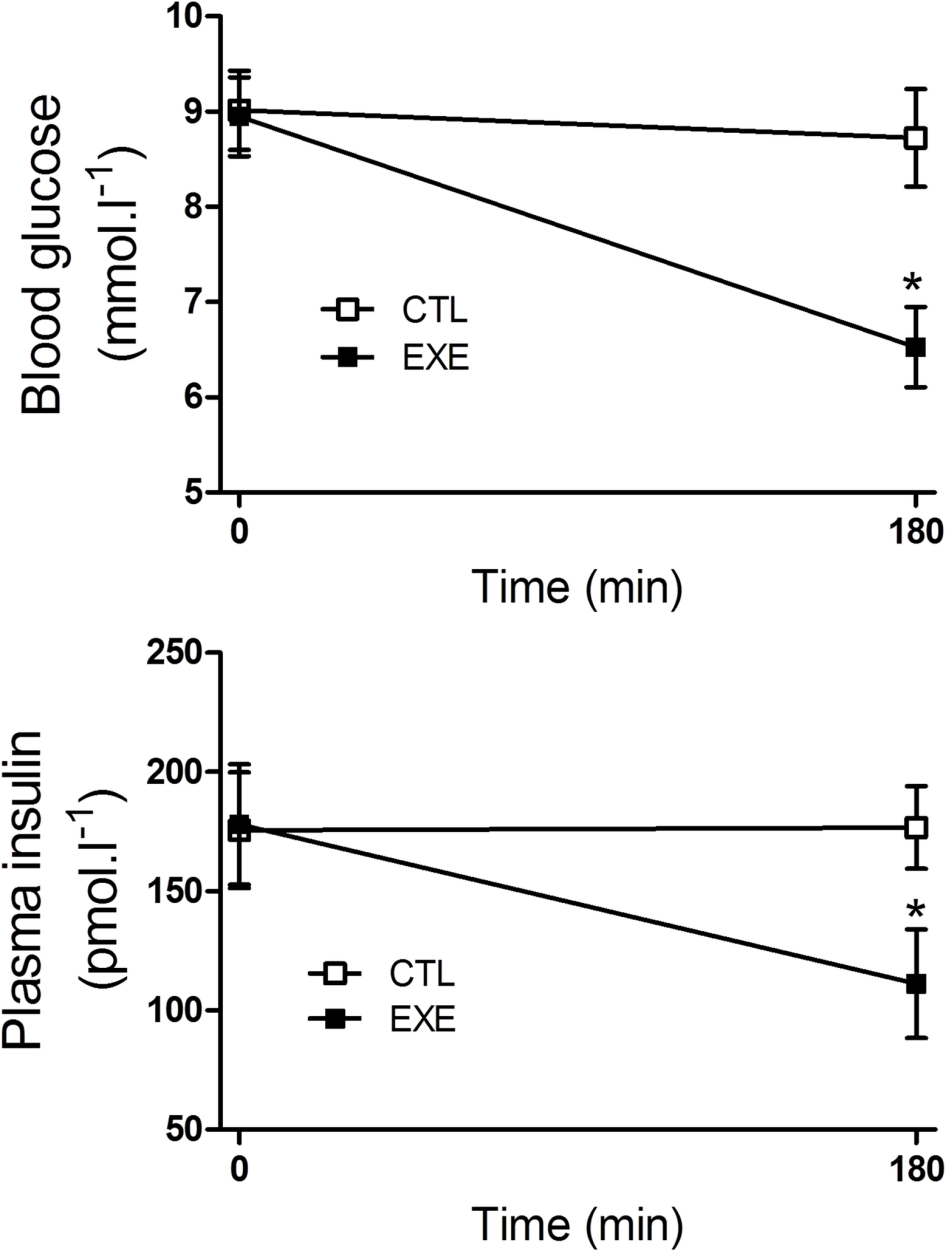
Acute exercise decreased plasma insulin and glucose. Blood glucose (A) and plasma insulin (B) were reduced in mice after a single bout of 3-h exercise on the treadmill. The data are presented as the mean ± S.E.M., n = 4–5. *p≤0.05 *vs* control.

After a single 3 h bout of exercise, the mice exhibited reduced glycemia (Fig 2A) and insulinemia (Fig 2B), compared with the control mice.

### Exercise increased insulin tolerance

**Fig 3:**
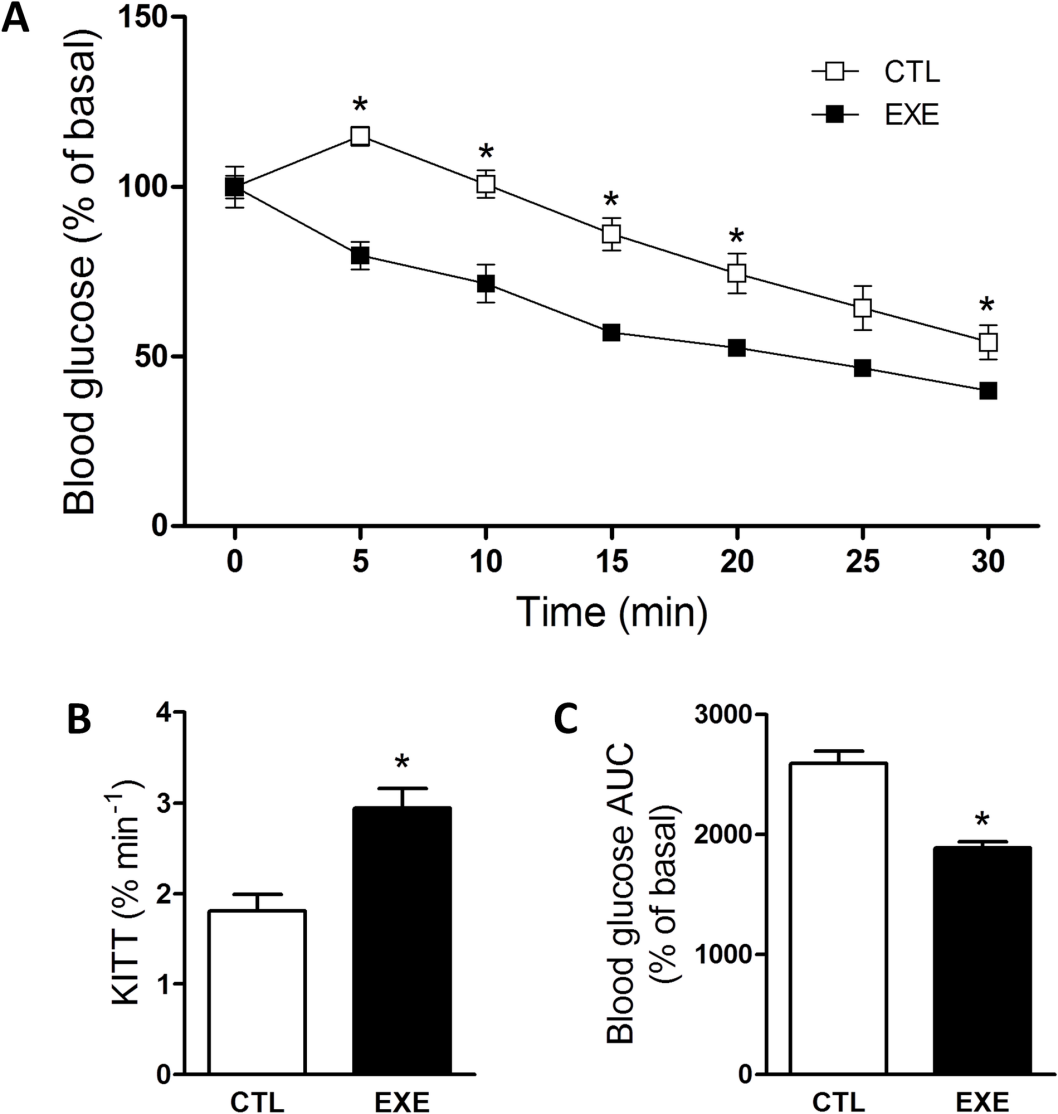
Acute exercise improved insulin tolerance. The ipITT (A), constant rate for the glucose decay (B) and AUC of glucose (C) of mice after a single bout of 3-h exercise on the treadmill. The data are presented as the mean ± S.E.M., n = 5. *p≤0.05 *vs* control.

To explain the apparent paradox of lower insulinemia and glycemia in exercised mice, we evaluated insulin response using the ipITT. After exercise, the mice were more responsive to insulin, as demonstrated by a lower ipITT value (Fig 3A) and a higher *k*_ITT_ value (Fig 3B). As shown by the area under the curve (AUC) of glucose values (Fig 3C), the increased insulin tolerance resulted in a lower glycemia during the test.

### Exercise increased the phosphorylation of AMPK-ACC but not IR-Akt

**Fig 4:**
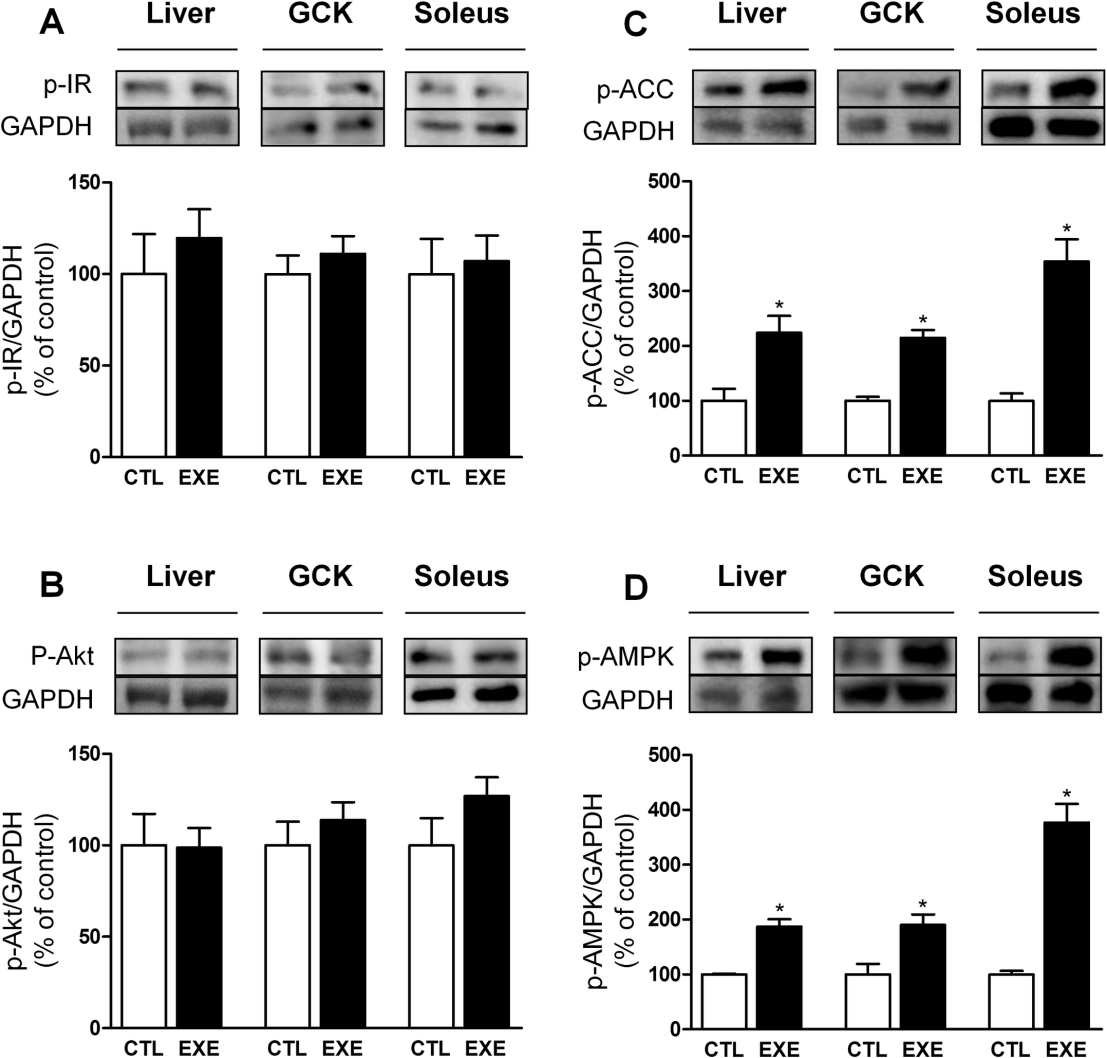
Acute exercise increased AMPK-ACC but not IR-AKT pathway activity. The p-IR (A) and p-AKT (B) protein levels were unaltered, whereas p-AMPK (C) and p-ACC (D) were increased in the liver, gastrocnemius (GCK) and soleus after a single bout of 3-h exercise on the treadmill. The data are presented as the mean ± S.E.M., n = 4–5. *p≤0.05 *vs* control.

The phosphorylation levels of IR (Fig 4A) and Akt (Fig 4B) were not changed, whereas phosphorylated AMPK (Fig 4C) and phosphorylated ACC (Fig 4D) were increased in the liver, soleus and gastrocnemius of mice, after exercise.

### Exercise increased GSIS from pancreatic islets

**Fig 5:**
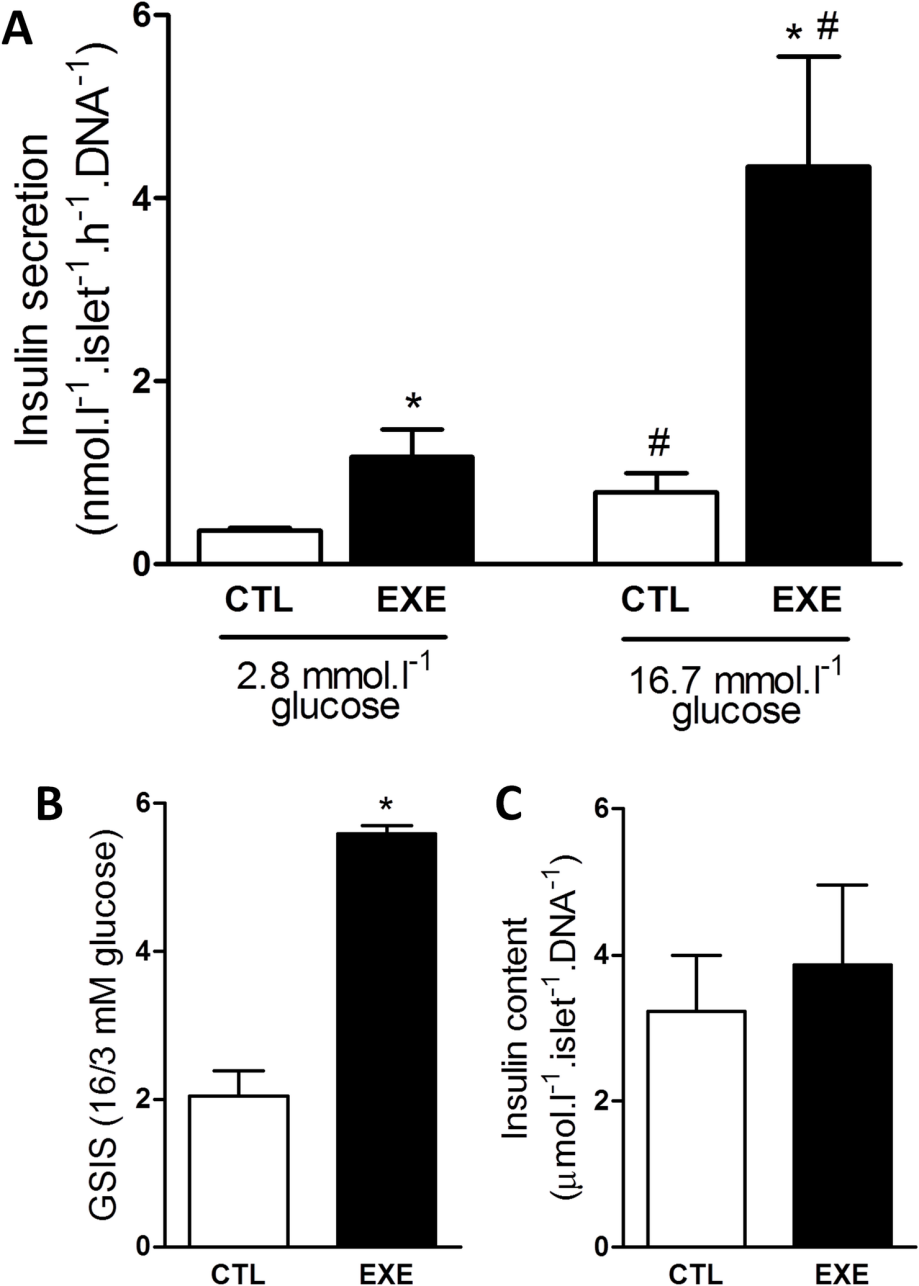
Acute exercise impaired insulin secretion and pancreatic islet function. A single bout of 3-h exercise on the treadmill reduced insulin secretion from isolated pancreatic islets under basal and stimulatory glucose conditions (A) and impaired pancreatic islets function as evaluated by GSIS (B) without affecting islets total insulin content (C). The data are presented as the mean ± S.E.M., n = 4. *p≤0.05 *vs* respective control and #p≤0.05 *vs* control 3 mmol.l^-1^ glucose.

Since the increased insulin sensitivity observed does not explain the lower concentration of insulin, in the plasma of exercised mice, we analyzed the GSIS of *ex vivo* pancreatic islets, isolated from the control and exercised mice. Exercise increased insulin secretion at sub- and supra-stimulatory glucose conditions (Fig 5A), and this insulin increase was accompanied by an increase in beta-cell function, as demonstrated by the higher GSIS (Fig 5B). Furthermore, exercise did not alter the total insulin content (Fig 5C).

### Exercise increased insulin clearance and decay

**Fig 6:**
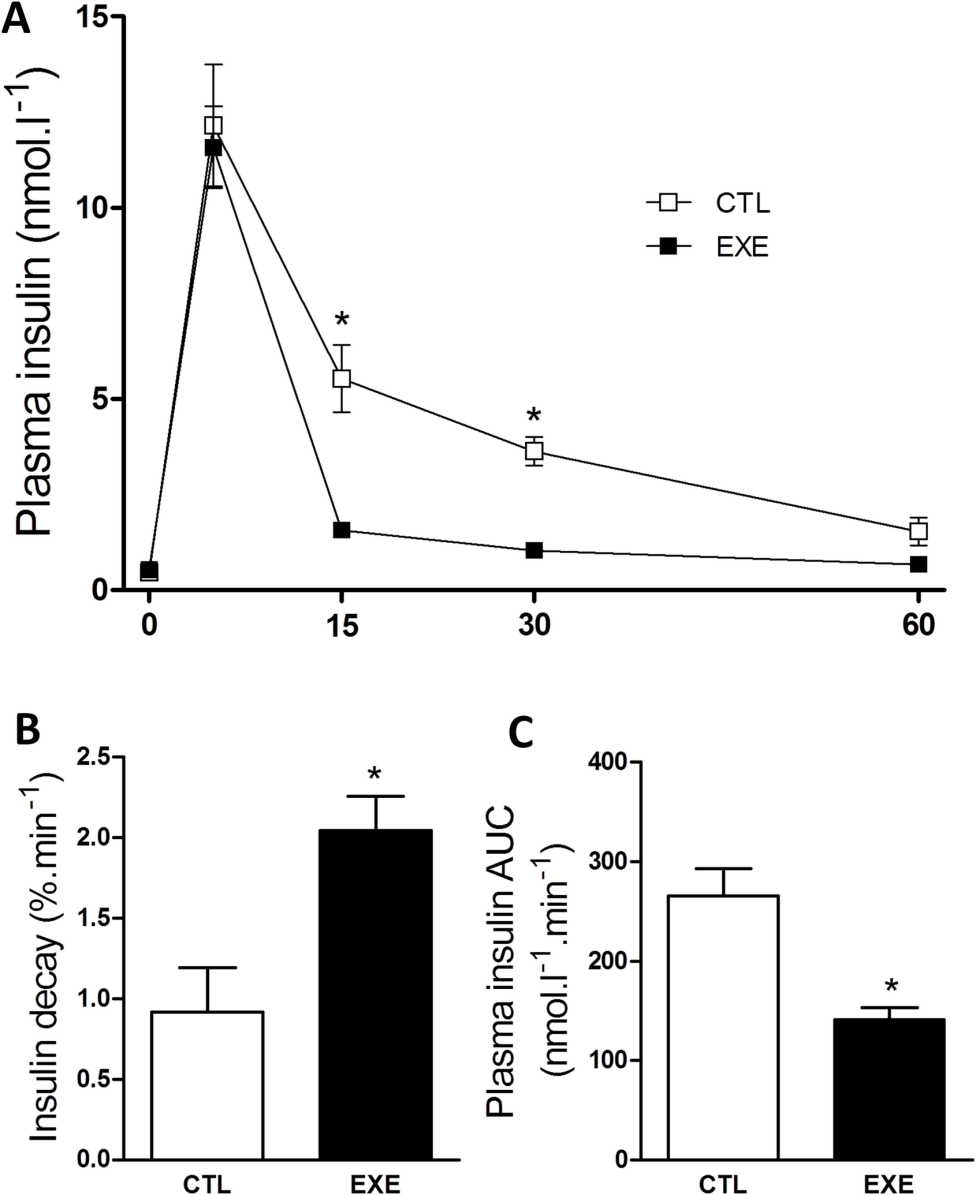
Acute exercise increased insulin clearance and decay. A single bout of 3-h exercise on the treadmill reduced insulin clearance (A), insulin decay rate (B) and AUC of insulin (C) during ipITT. The data are presented as the mean ± S.E.M., n = 3–4. *p≤0.05 *vs* control.

Because the increased GSIS does not explain the reduction in insulinemia, we evaluated insulin clearance in the exercised mice. We found that exercise increased insulin clearance (Fig 6A) and the insulin decay rate after insulin administration (Fig 6B), resulting in lower insulin levels during ITT, as judged by the AUC of insulin (Fig 6C). These results explain the reduced insulinemia in exercised mice.

### Exercise increased IDE expression in liver and skeletal muscle

**Fig 7:**
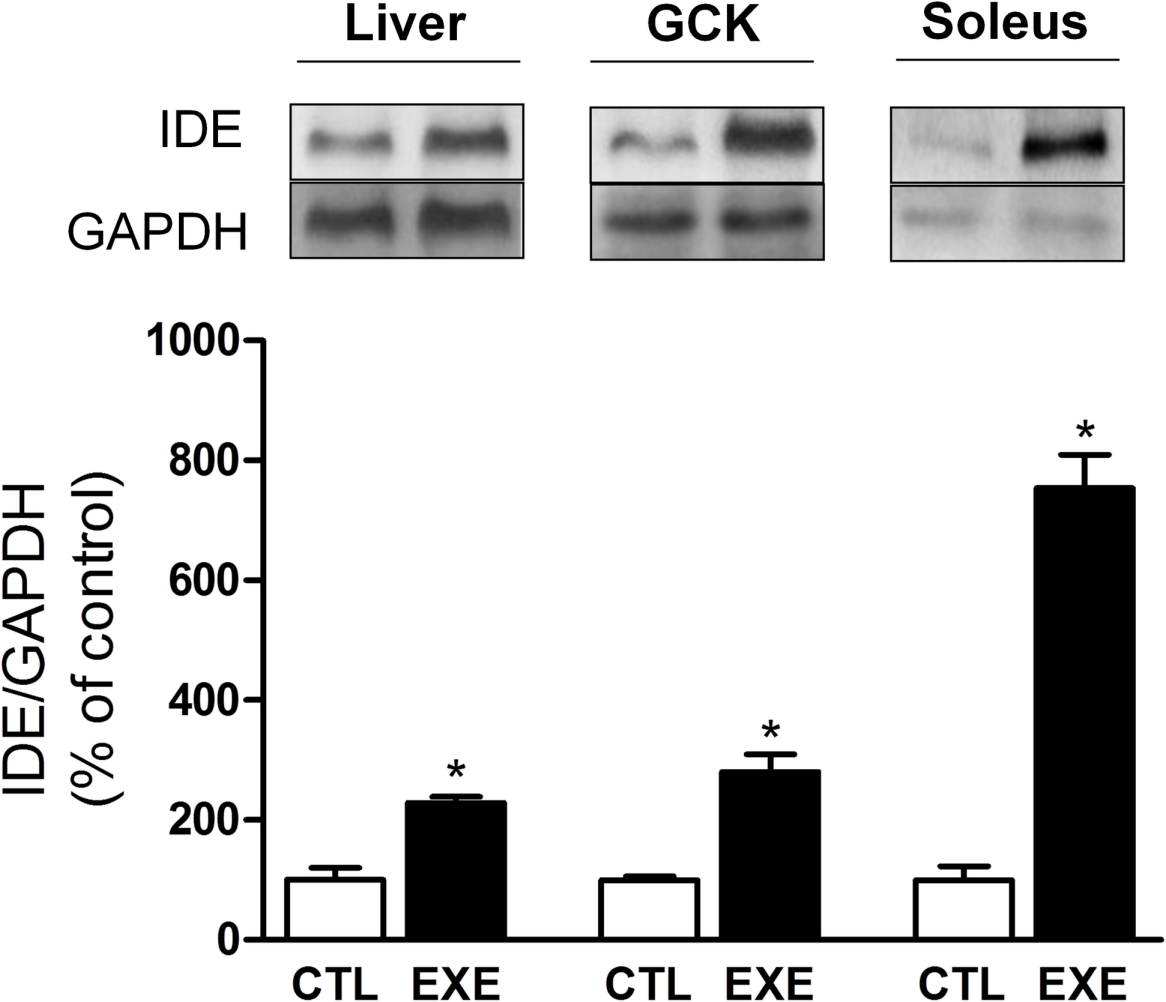
Acute exercise increased IDE expression. A single bout of 3-h exercise on the treadmill increased IDE protein levels in the liver (approximately 2×), gastrocnemius (approximately 2×) and soleus (approximately 8×) of mice. The data are presented as the mean ± S.E.M., n = 4–5. *p≤0.05 *vs* control.

We also investigated the mechanism by which insulin clearance was reduced in response to exercise. IDE protein levels were increased in the liver, soleus and gastrocnemius tissues (Fig 7), which indicates that insulin clearance in mice is likely to be increased as a result of increased IDE expression.

### AMPK activation increases IDE expression in C2C12, but not in HEPG2 cells

**Fig 8:**
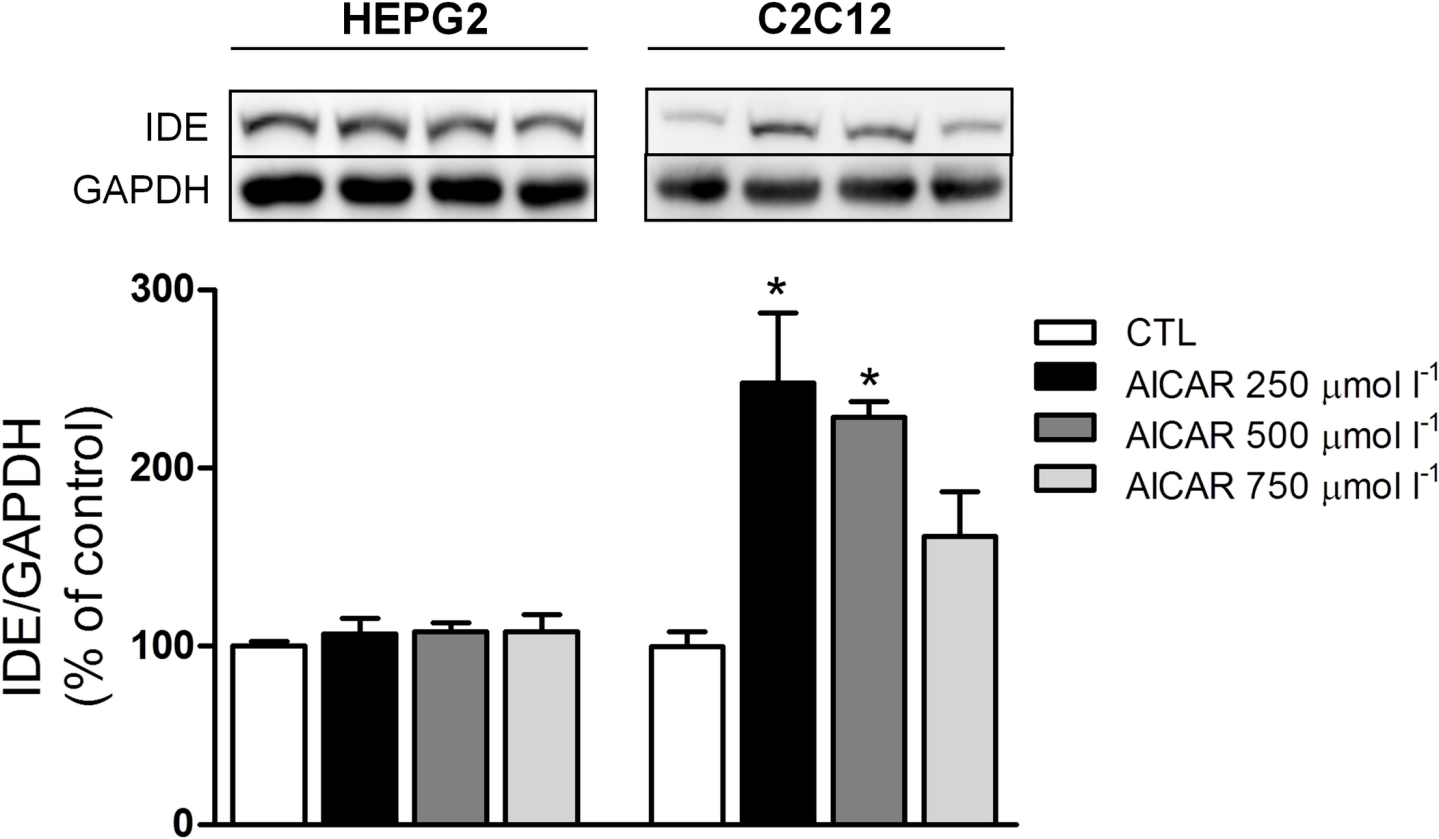
AICAR increases the IDE expression in C2C12 cells, but not in HEPG2 cells. The IDE protein levels did not change in HEPG2 cells after 3-h treatment at 250, 500 and 750 μmol l-1 AICAR (A), but it increased in C2C12 cells after 3-h treatment at 250 and 500 μmol l-1 AICAR (B). The data are presented as the mean ± S.E.M., n = 3–4. *p≤0.05 *vs* control.

Finally, we evaluated whether activation of AMPK-ACC pathway could increase the IDE expression. In HEPG2 cells, 3-h of treatment with different concentrations of AICAR did not change the IDE expression (Fig 8A). However, in C2C12 cells, 3-h incubation at 250 and 500 μmol l^-1^ AICAR, significantly increased IDE expression (Fig 8B). These data indicate that increased IDE expression in the skeletal muscle, in response to exercise, could be mediated by activation of AMPK-ACC pathway.

## Discussion

The effects of physical exercise on insulin clearance and IDE expression, in healthy lean subjects, are not yet fully elucidated. In the literature there is evidence either in favor to an increase [27, 31–33], or to a decrease [39] in insulin clearance, induced by exercise. Here, we found that a single bout of exercise reduces insulinemia in lean mice, mainly by increase insulin clearance, probably due to an augmentation of the IDE expression in the liver and skeletal muscle. We suppose that this augmentation in the IDE expression **is** mediated by activation of AMPK-ACC pathway in the skeletal muscle, but not in the liver. We believe that the increase in insulin clearance and IDE expression contribute to avoid a hazardous decrease in glycemia during the physical exercise, turning it safer and effective.

It is known that exercise reduces plasma insulin concentration [40, 41] and this effect is explained, at least in part, by decrease in insulin secretion [42, 43]. However, no alteration [44] or even increase in the insulin release, after exercise, were observed [45–47]. Corroborating these last studies, we found an increased GSIS in isolated pancreatic islet from exercised mice (Fig 5). We speculate that this increase in secretion is due to a cross-talk between the skeletal muscle and pancreas, mediated by myokines [48], particularly interleukin-6 (IL-6) [47, 49]. Irrespective to the mechanism, increased insulin secretion does not explain the lower insulinemia found in our exercised mice.

The insulin concentration in the plasma depends on the balance between insulin secretion and clearance. Therefore, the increased insulin clearance observed (Fig 6) could explain why the insulinemia is reduced in mice after a single bout of exercise, despite an increased GSIS. In agreement, some studies also demonstrated that acute [46] and chronic [31, 33] exercise induce an increase of the insulin clearance. However none of these studies measured the IDE expression.

Insulin clearance is primarily dependent on degradation of insulin, which is mediated mainly by IDE in the liver [3]. In lean trained Swiss mice, rested for 24 hours, the reduction in insulin clearance was attributed to a lower IDE expression in the liver, despite a higher IDE expression in the skeletal muscle [39]. In this line, and corroborating previous work [29], we observed here that the expression of IDE was increased by two-fold in the liver of exercised mice, explaining, at least in part, the augmented insulin clearance. Interestingly, IDE was also increased in the soleus (by eight-fold) and gastrocnemius (by two-fold) skeletal muscles of exercised mice. These results were intriguing, considering that the skeletal muscle is not the primary organ responsible for insulin degradation. However, during exercise, blood flow is significantly increased in the skeletal muscle. Thus, this tissue could play an important role in the insulin removal and degradation during physical activity. In fact, in diet-induced obese mice, an expressive increase in IDE activity was observed only in acute exercised muscle [18].

In diet-induced obese mice, which display hyperinsulinemia, acute exercise also increases insulin clearance and IDE expression, normalizing the insulinemia, and this could be an important beneficial effect of acute exercise, in diseases related to insulin resistance. By contrast, lean mice do not display hyperinsulinemia, and we believe that the increase in IDE expression and insulin clearance, in these mice, are important for the effectiveness and safety of physical exercise. During and immediately after intense exercise, a substantial insulin-independent increase in glucose uptake by skeletal muscle is observed [50–52]. This increased uptake, associated with increased insulin secretion by the islets, would provoke a fast and hazardous decrease in glycemia. However, a significant increase in the degradation of insulin, by the active skeletal muscle, could prevent the depletion of the glucose stock from the plasma.

Although the effect of exercise on insulin clearance in lean subjects needs more investigations, it is well known that exercise improves insulin sensitivity [53] and reduces glycemia [54, 55]. Here, despite an increased insulin tolerance (Fig 3), acute exercise *per se* did not activate the canonical insulin signaling pathway (IR-Akt) (Fig 4), in agreement with previous data [56, 57]. In fact, acute exercise increased the activity of the AMPK-ACC pathway in the skeletal muscle (Fig 4), corroborating previous reports [58, 59].

Finally, we investigated if activation of AMPK-ACC pathway contributes to the increase in IDE expression. Interestingly, an increased IDE expression was noticed in C2C12, but not in HEPG2 cells, after exposure to AICAR for 3-h (Fig 8). Thus, activation of AMPK, by exercise, could contribute to the increase of IDE expression in the skeletal muscle. It is of note that IDE can also degrade receptor-bound insulin [60] interrupting IR phosphorilation and activation. Thus, higher activity of the AMPK-ACC pathway could block the insulin signaling, *via* increase IDE expression, corroborating our hypothesis that increase in the degradation of insulin, by the active skeletal muscle, would protect against hypoglycemia during physical activities.

In conclusion, we present evidence that a single bout of exercise increases insulin clearance to maintain glycemia at or close to physiological levels. This effect may occur *via* an increase in the expression of IDE in the liver and skeletal muscle, which may have implications for the effectiveness and safety of physical exercise in lean mice. Our data also suggest that activation of AMPK could be the mechanism whereby acute exercise increases IDE expression in the skeletal muscle.

## Supporting Information

S1 Fig VO_2_ max test. The treadmill exercise for VO_2_ max test included a warm-up period of 5 min at 10 cm sec^-1^ with subsequently increase in the treadmill speed by 5 cm sec^-1^ each minute, until the mice reached exhaustion (for details, see Materials and Methods). The data are presented as the mean ± S.E.M., n = 10.
(TIF)

S1 Table VO_2_ max test data. VO_2_ max, maximum speed and run distance reached by the mice (1–10) during the VO_2_ max test. The mean data are presented as the mean ± S.E.M., n = 10.
(DOCX)
