# Supplementary figures and images for: Acute Exercise Improves Insulin Clearance and Increases the Expression of Insulin-Degrading Enzyme in the Liver and Skeletal Muscle of Swiss Mice
Source: PLoS One. 2016 Jul 28;11(7):e0160239. doi: 10.1371/journal.pone.0160239 (PMC4965115; doi:10.1371/journal.pone.0160239)

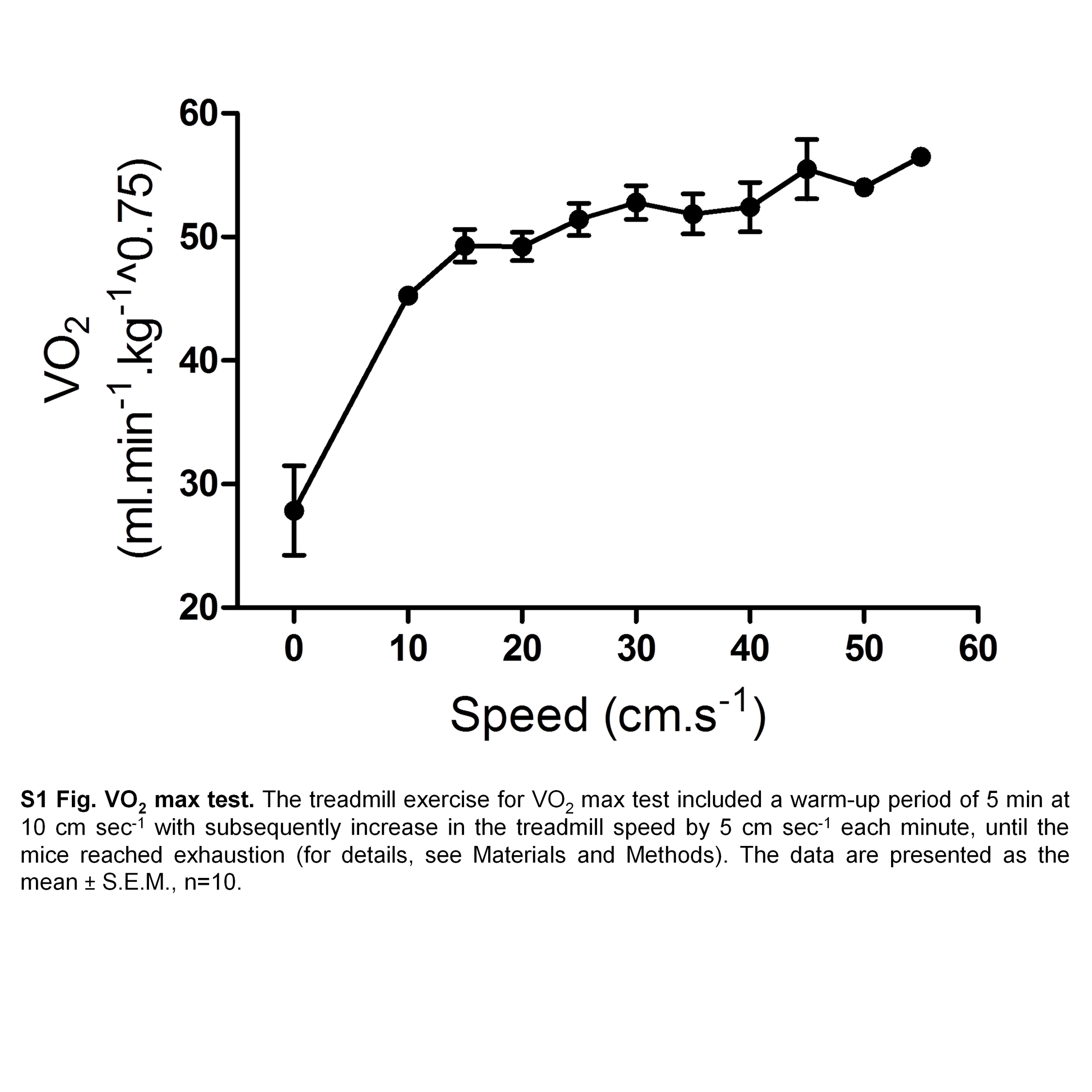

Supplement: S1 Fig — The treadmill exercise for VO2 max test included a warm-up period of 5 min at 10 cm sec-1 with subsequently increase in the treadmill speed by 5 cm sec-1 each minute, until the mice reached exhaustion (for details, see Materials and Methods). The data are presented as the mean ± S.E.M., n = 10. (TIF) [file pone.0160239.s001.tif]
