# Supplementary material for: Acute Exercise Improves Insulin Clearance and Increases the Expression of Insulin-Degrading Enzyme in the Liver and Skeletal Muscle of Swiss Mice
Source: PLoS One. 2016 Jul 28;11(7):e0160239. doi: 10.1371/journal.pone.0160239 (PMC4965115; doi:10.1371/journal.pone.0160239)
Supplement: S1 Table — VO2 max, maximum speed and run distance reached by the mice (1–10) during the VO2 max test. The mean data are presented as the mean ± S.E.M., n = 10. (DOCX) [file pone.0160239.s002.docx]

**S1 Table. VO_2_ max test data.**

| Mice | VO_2_ max (ml/min/kg^0.75) | Maximum speed (cm/s) | Run distance (cm) |
| --- | --- | --- | --- |
| 1 | 56.476 | 55 | 18550 |
| 2 | 54.754 | 45 | 12860 |
| 3 | 58.929 | 35 | 8340 |
| 4 | 50.087 | 35 | 9135 |
| 5 | 53.503 | 30 | 6930 |
| 6 | 57.232 | 30 | 7590 |
| 7 | 55.711 | 35 | 9030 |
| 8 | 59.975 | 45 | 14745 |
| 9 | 56.272 | 30 | 7440 |
| 10 | 49.81 | 35 | 10430 |
| Mean | **55.27 ± 1.065** | **37 ± 2.261** | **10505 ± 1191** |
